# Rapamycin Treatment of Tendon Stem/Progenitor Cells Reduces Cellular Senescence by Upregulating Autophagy

**DOI:** 10.1155/2021/6638249

**Published:** 2021-02-01

**Authors:** Daibang Nie, Jianying Zhang, Yiqin Zhou, Jiuyi Sun, Wang Wang, James H.-C. Wang

**Affiliations:** ^1^Department of Immunology, College of Basic Medicine, Chongqing Medical University, Chongqing, China; ^2^MechanoBiology Laboratory, Department of Orthopaedic Surgery, University of Pittsburgh School of Medicine, Pittsburgh, PA, USA; ^3^Department of Joint Surgery and Sports Medicine, Shanghai Changzheng Hospital, Second Military Medical University, Shanghai, China; ^4^Department of Orthopaedics, Navy Medical Center of PLA, Shanghai, China; ^5^Department of Physical Medicine and Rehabilitation, University of Pittsburgh, Pittsburgh, PA, USA; ^6^Department of Bioengineering, University of Pittsburgh, Pittsburgh, PA, USA

## Abstract

The elderly population is prone to tendinopathy due to aging-related tendon changes such as cellular senescence and a decreased ability to modulate inflammation. Aging can render tendon stem/progenitor cells (TSCs) into premature senescence. We investigated the effects of rapamycin, a specific mTOR inhibitor, on the senescence of TSCs. We first showed that after treatment with bleomycin in vitro, rat patellar TSCs (PTSCs) underwent senescence, characterized by morphological alterations, induction of senescence-associated *β*-galactosidase (SA-*β*-gal) activity, and an increase in p53, p21, and p62 protein expression. Senescence of PTSCs was also characterized by the elevated expression of MMP-13 and TNF-*α* genes, both of which are molecular hallmarks of chronic tendinopathy. We then showed that rapamycin treatment was able to reverse the above senescent phenotypes and increase autophagy in the senescent PTSCs. The activation of autophagy and senescence rescue was, at least partly, due to the translocation of HMGB1 from the nucleus to the cytosol that functions as an autophagy promoter. By reducing TSC senescence, rapamycin may be used as a therapeutic to inhibit tendinopathy development in the aging population by promoting autophagy.

## 1. Introduction

Tendinopathy is a prevalent tendon disorder that affects millions of Americans and costs billions of healthcare dollars every year. Aging is thought to be one of the major risk factors for tendinopathy, which is characterized by abnormal thickening, degeneration, and calcification of tendon tissues [1, 2]. Aging decreases the ability of the tendon to maintain its structural integrity, largely due to the loss of the stem cell population within the tendon. The tendon stem/progenitor cell (TSC) population plays a major role in tendon homeostasis and repair by replacing lost mature tenocytes. TSCs are also considered to contribute to the pathogenesis of tendinopathy [3]. Recent studies have demonstrated that the frequency and proliferation rates of TSCs are both reduced, and cell cycle progression is delayed in aged TSCs, which may impair tendon regeneration capacity and lead to age-related tendinopathy [1, 4]. Although it is known that the potentials of TSC proliferation and differentiation decline with aging, the mechanisms regulating TSC aging are poorly understood.

Stem cell numbers and activities are regulated by a variety of mechanisms, including proliferation and differentiation, apoptosis, and senescence [5]. The progressive loss of these cellular mechanisms accounts for the dysfunction of stem cells. Cells enter the state of senescence when they can no longer divide in presence of abundant growth factors. The key feature of senescent cells is the expression of the senescence-associated secretory phenotype (SASP). Typical characteristics of cellular senescence include enlarged cell size with abnormal morphology and resistance to apoptosis. Additionally, upregulation of the senescence-associated *β*-galactosidase (SA-*β*-gal) activity and p21 and/or p16^Ink4a^-mediated irreversible cell-cycle arrest are molecular indicators of senescence [5]. Importantly, it has been demonstrated that the number of senescent cells is positively associated with normal aging in various organs and tissues, including tendon [6–9].

Senescence can be induced as a result of DNA damage, supported by the promotion of senescent markers with typical damage inducers such as telomere dysfunction, oxidative stress, and UV [10]. Similarly, studies have found that bleomycin, which causes DNA lesions by single and double strand breaks, induces senescence in alveolar epithelial cells and human fibroblasts [11, 12]. On the other hand, senescence has been shown to be prevented by autophagy, which in turn contributes to the maintenance of cell “stemness” over time [13, 14]. Autophagy exhibits fundamental importance in cellular homeostasis and tissue remodeling during normal development, and it is crucial for stem cell maintenance and self-renewal. Moreover, autophagy has been implicated in regulating the differentiation of stem cells and their progenies [15]. Recent studies have highlighted the central role of autophagy in balancing the dormancy, self-renewal, and plasticity of stem cells, and the activities of autophagy can be quantitatively monitored by measuring the levels of autophagy markers, such as microtubule-associated protein 1A/1B-light chain 3 (LC3), and p62/SQSTM1 [16].

Autophagy is regulated by a number of signal transduction pathways, among which, a tight, inverse correlation of autophagy induction and the mammalian target of rapamycin (mTOR) activation have been well established [17–19]. The serine/threonine protein kinase mTOR is an important regulator of multiple cellular functions. During chronic inflammation, mTOR controls the proliferation of both immune and nonimmune cells, and it promotes the expression of cytokines and chemokines to determine cell fate, migration, and invasion. mTOR can also facilitate remodeling of the extracellular matrix, leading to the occurrence of fibrosis in the inflamed area [20]. Given such functional significance of mTOR, rapamycin, a potent mTOR antagonist that has been reported to suppress interleukin-2- (IL-2-) stimulated T cell proliferation and confer immunosuppression, has been developed for its important therapeutic prospects. Rapamycin was first approved in 1999 for allograft protection in kidney transplant recipients and approved in 2002 for the antirestenosis aspect for patients with coronary artery repairs [21]. In recent years, interests of rapamycin have been drawn to its potential as an anticancer drug, as well as its beneficial effects shown in a number of animal models of neurodegeneration and osteoarthritis [22, 23]. Moreover, researchers have found the association of the mTOR pathway signaling activity with cellular and organismal aging [24]. As the antagonist of mTOR, rapamycin retards multiple aspects of aging in mice and decelerates senescence in normal and progeric human cells [25–27]. Therefore, the mTOR pathway may interconnect cellular senescence, organismal aging, and diseases associated with aging.

Many senescence inducers such as DNA damage and reactive oxygen species (ROS) have been shown to induce the cellular stress response. These “danger” signals can be sensed by a group of fast-reacting endogenous damage-associated molecular pattern (DAMP) molecules, which will lead to temporal and spatial regulation of signal transductions that will decide the cell fate [28]. High mobility group box 1 (HMGB1), a conserved nuclear protein, has been shown to play subcellular localization-dependent roles upon stress [29]. To our interest, the cytosolic HMGB1 has been reported to induce autophagy [30]. Therefore, this study is aimed at studying the relationship between senescence and autophagy in rat TSCs, by assessing the effect of bleomycin and rapamycin treatment and addressing the molecular mechanisms involved. Our data showed that rapamycin can decrease TSC senescence, likely via upregulation of autophagy.

## 2. Materials and Methods

### 2.1. Patellar Tendon Stem Cell (PTSC) Isolation and Culture

PTSCs were isolated from the patellar tendons of female Sprague-Dawley (SD) rats (5 months) based on a previously published protocol [31]. The protocol for use of the rats was approved by the IACUC of the University of Pittsburgh. Briefly, tendon sheaths from the patellar tendons were removed to obtain the core portions of the tendons. Tendon samples were then minced into small pieces, and each 100 mg wet tissue sample was digested in 1 ml phosphate buffered saline (PBS) containing 3 mg collagenase type I (GIBCO) and 4 mg dispase (GIBCO) at 37°C for 2 h. After centrifugation at 3500 rpm/min for 15 min to remove the enzymes, the cells were cultured in growth medium consisting of Dulbecco's Modified Eagle's Medium (DMEM; Lonza) supplemented with 20% fetal bovine serum (FBS; Gemini Bio-Products), 100 U/ml penicillin, and 100 *μ*g/ml streptomycin (Thermo Scientific) at 37°C with 5% CO_2_. The medium was changed every 3 days, and cells were split when over 90% confluence was reached. In all further culture experiments, PTSCs at passage 2 were used.

### 2.2. Bleomycin and Rapamycin Treatment of TSCs

For all treatments, rat PTSCs were plated at a density of 5 × 10^4^ cells/well in a 12-well cell culture plate in regular growth media. After the cells were fully attached, the media were replaced with drug-containing growth media with reduced FBS (10%). Specifically, for the bleomycin treatment, cells were treated with 10 *μ*g/ml or 50 *μ*g/ml bleomycin (Millipore) at 37°C with 5% CO_2_ for 5 days, and growth media with reduced FBS (10%) were used as control. For the rapamycin treatment, cells were treated with different concentrations of rapamycin (0.1 nM, 1 nM, 10 nM, 25 nM, 50 nM, and 100 nM, Sigma) at 37°C with 5% CO_2_ for 3 days, and DMSO was used as control. For the bleomycin and rapamycin treatment, cells were treated with 50 *μ*g/ml bleomycin and 25 nM rapamycin at 37°C with 5% CO_2_ for 5 days, with DMSO as control.

### 2.3. Senescence-Associated *β*-Galactosidase (SA-*β*-gal) Staining

*β*-Galactosidase-positive cells were detected by the Senescent Cells Histochemical Staining Kit (Sigma-Aldrich). Briefly, the monolayer of cells was washed twice with PBS prior to a fixation of 6 to 7 min, followed by another wash of three times with PBS. Then, staining mixture was added, and the cells were incubated at 37°C overnight. After incubation, the cells were photographed on an inverted microscope (Nikon Eclipse, TE2000-U).

### 2.4. Western Blot Analysis

The cells were lysed using RIPA lysis buffer (Sigma) with 1 mM PMSF (Sigma), according to the suggested procedure from the manufacturer. All the samples were measured for total protein concentration using a BCA Protein Assay Kit (Thermo Scientific) to ensure equal loading. Loading buffer was added to 30 *μ*g protein and separated on 10% SDS-PAGE gels before transferring onto PVDF membranes (Bio-Rad). The blots were blocked using 5% nonfat dry milk (Bio-Rad) at room temperature for 1 h with gently shaking and then incubated for overnight with primary antibodies against LC3A/B (Cell Signaling, 1 : 1000), SQSTM1/p62 (Cell Signaling, 1 : 1000), p-S6 (Cell Signaling, 1 : 1000), S6 (Cell Signaling, 1 : 1000), p53 (Cell Signaling, 1 : 1000), p21 (Abcam, 1 : 1000), HMGB1 (Abcam, 1 : 1000), Histone H3 (Cell Signaling, 1 : 2000), and *β*-actin (Abcam, 1 : 10000). Corresponding secondary antibody (LI-COR Biosciences, 1 : 15000) incubation was carried out the next day for 1 h at room temperature, with three washes in-between incubations with PBS-T buffer. Then, the protein bands were visualized by the LiCoR Odyssey imager (LI-COR Biosciences). Semiquantification was performed using NIH ImageJ software.

### 2.5. Quantitative Real-Time Reverse Transcription Polymerase Chain Reaction (qRT-PCR)

Total RNA was extracted using a RNeasy Mini Kit (Qiagen). First-strand cDNA was synthesized in a 20 *μ*l reaction volume from 1 *μ*g total RNA by reverse transcription with SuperScript II (Invitrogen). The conditions for the cDNA synthesis were 65°C for 5 min and cooling for 1 min at 4°C, then 42°C for 50 min, and 72°C for 15 min. The qRT-PCR was carried out using QIAGEN QuantiTect SYBR Green PCR Kit (Qiagen). Rat-specific primers used for RT-PCR were TNF-*α*: 5′-AAA TGG GCT CCC TCT CAT CAG TTC-3′ (forward), 5′-TCT GCT TGG TGG TTT GCT ACG AC-3′ (reverse); IL-6: 5′-TCC TAC CCC AAC TTC CAA TGC TC-3′ (forward), 5′-TTG GAT GGT CTT GGT CCT TAG CC-3′ (reverse), and MMP-13: 5′-CTG ACC TGG GAT TTC CAA AA-3′ (forward), 5′-ACA CGT GGT TCC CTG AGA AG-3′ (reverse) [32, 33]. GAPDH was used as an internal control. All primers were synthesized by Invitrogen. After an initial denaturation for 10 min at 95°C, PCR was performed for 30 cycles for GAPDH and 40 cycles for TNF-*α*, IL-6, and MMP-13, with denaturation for 50 seconds at 95°C at each cycle and annealing for 30 seconds at 58°C. Relative expression levels of each gene were detected at least three times independently. Data were analyzed by the 2^−*ΔΔ*Ct^ method.

### 2.6. Immunofluorescence Analysis of HMGB1 Translocation

Cells were seeded in a 4-well Chamber Slide (Thermo Scientific), treated with 25 nM rapamycin or DMSO for 12 h, or stimulated with HBSS alone (Thermo Scientific) for 3 h, and each group was recovered in growth media with reduced FBS (10%) for 12 h. And then, the cells were washed twice in PBS and fixed with 4% paraformaldehyde for 15 min at room temperature. The fixed cells were treated with 0.1% of Triton X-100 for 15 min, then washed with PBS for another three times. After blocking with 2% bovine serum albumin (BSA) for 1 hr at room temperature, the cells were incubated with rabbit anti-rat HMGB1 antibody (Abcam, 1 : 250) overnight at 4°C. After washing the cells with PBS, Cy3-conjugated goat anti-rabbit secondary antibody (1 : 500, Invitrogen Molecular Probes) was applied for 1 hr at room temperature. The cells were also counterstained with H33342 staining (Sigma). The stained cells were imaged using Nikon A1 confocal microscopy (Nikon).

### 2.7. Cell Proliferation Assay

Cell proliferation was determined by the Cell Counting Kit-8 (CCK-8) assay according to manufacturer's instruction (Sigma). Briefly, 100 *μ*l culture medium (DMEM+10% FBS) containing 3 × 10^3^ cells was seeded in a 96-well plate and incubated at 37°C for 24 h to allow attachment. Then, the medium was replaced with fresh culture medium containing rapamycin (Millipore) at indicated concentrations. After 72 h culture, the medium was removed, and 10 *μ*l of CCK-8 solution with 100 *μ*l culture medium was added into each well and incubated at 37°C for another 2 hours. The absorbance was recorded at 450 nm using a microplate reader (SpectraMax M5, Molecular Devices). Additionally, cell proliferation was assessed by standard cell count with a hemocytometer.

### 2.8. Statistical Analysis

One-way analysis of variance (ANOVA) was used, followed by Fisher's LSD post hoc test for multiple comparisons. All statistical tests were done using GraphPad Prism 7 (GraphPad Software). Differences with a *p* value less than 0.05 were considered as statistically significant.

## 3. Results

### 3.1. Bleomycin Induces PTSC Senescence

**Figure 1:**
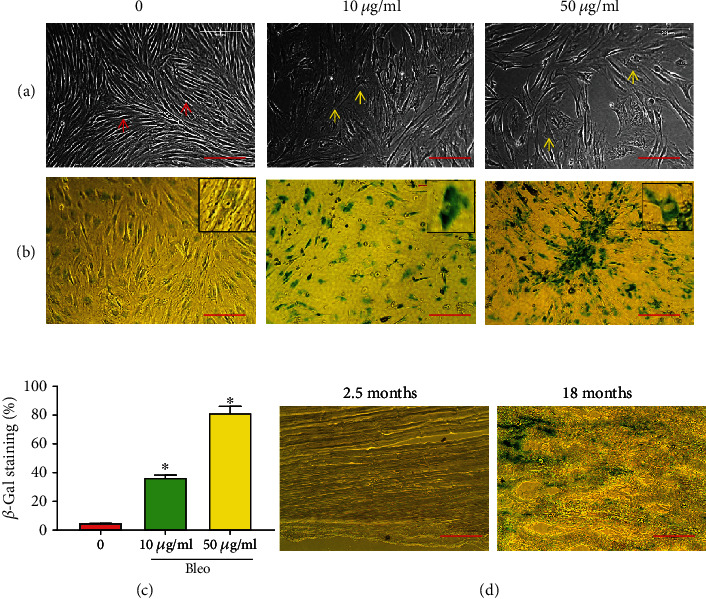
Bleomycin induces PTSC senescence in a dose-dependent manner. (a) Rat PTSCs were treated without or with bleomycin (Bleo) for 5 days. (b) PTSCs were stained for senescence-associated *β*-galactosidase (*β*-gal) activity. As seen, at 10 and 50 *μ*g/ml, the senescent cells look flattened and enlarged (a, yellow arrows) compared to control cells, which are elongated (a, red arrows). Scale bar = 200 *μ*m. (c) Semiquantification shows a marked increase in the number of *β*-gal-positive cells in a bleomycin-dose dependent manner. Results are expressed as mean ± SD, *n* = 3. ^∗^*p* < 0.01 compared to the control group. (d) Tendon tissues obtained from 2.5 month-old and 18 month-old rats were stained for the *β*-gal activity. It is evident that the young tendon is well organized without staining of *β*-gal; in contrast, aged tendon is disorganized and stained with *β*-gal.

When rat PTSCs were treated with various concentrations of bleomycin for 5 days, they started to exhibit a typical morphology for cellular senescence, indicated by the change of cell shape into a distinct, enlarged, and flat phenotype (Figure 1(a)). This morphological alteration was accompanied with increasing senescence-associated *β*-gal (SA-*β*-gal) activity (Figure 1(b)) and in a dose-dependent manner (Figure 1(c)). Specifically, over 80% of the rat, PTSCs demonstrated a gain of the SA-*β*-gal activity after exposed to 50 *μ*g/ml of bleomycin for 5 days (Figures 1(b) and 1(c)). When we compared the tendon tissues from young (2.5 months) and aged (18 months) rats, we found similar SA-*β*-gal activity to that observed from the in vitro staining of PTSCs. Also, aged tendon exhibited apparent tissue degeneration in comparison with the young tendon (Figure 1(d)). These results showed that bleomycin treatment induces cellular senescence in rat PTSCs; hence, bleomycin treatment of PTSCs can be used as an experimental model to investigate TSC senescence in vitro.

### 3.2. Rapamycin Decreases PTSC Senescence

**Figure 2:**
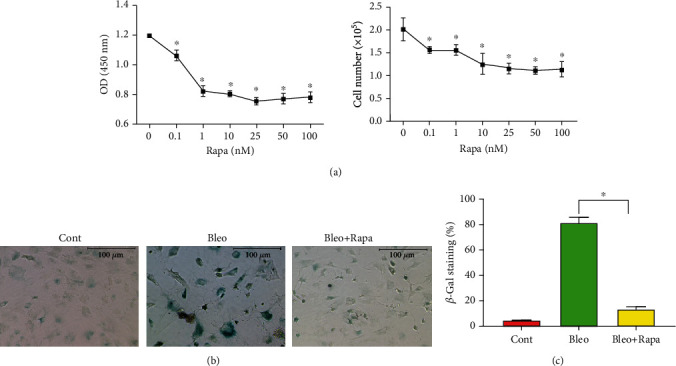
Rapamycin decreases PTSC senescence. (a) Treatment of PTSCs with rapamycin reduces cell proliferation. Data are presented as the mean ± SD, triplicates. ^∗^*p* < 0.01 compared to the control group without rapamycin treatment. (b) Treatment of PTSCs with 50 *μ*g/ml bleomycin induces *β*-gal, but it is abolished by combined treatment with 25 nM rapamycin for 5 days. Scale bar = 100 *μ*m. (c) Semiquantification confirms that rapamycin blocks *β*-gal induced by bleomycin. Results are expressed as mean ± SD, *n* = 3. ^∗^*p* < 0.01, compared to the bleomycin treatment group. Cont: control; Bleo: bleomycin; Rapa: rapamycin.

We investigated whether the bleomycin-induced cellular senescence can be prevented by autophagy. We first tested the sensitivity of the PTSC to various doses of rapamycin, the classic autophagy activator. We found a dose-dependent reduction in the stem cell growth (Figure 2(a)). Nonetheless, rapamycin was well tolerated by PTSCs. When they were treated with 25 nM rapamycin in combination with the senescence inducer bleomycin, cell morphology was preserved, which was otherwise altered by bleomycin (Figure 2(b)). Also, rapamycin markedly decreased the SA-*β*-gal activity, as another feature associated with cellular senescence (Figure 2(b)). Although it was not a complete rescue relative to the nontreated control, semiquantification analysis confirmed that rapamycin could significantly reduce TSC senescence caused by bleomycin (Figure 2(c)).

### 3.3. Rapamycin Decreases PTSC Senescence by Upregulating Autophagy

**Figure 3:**
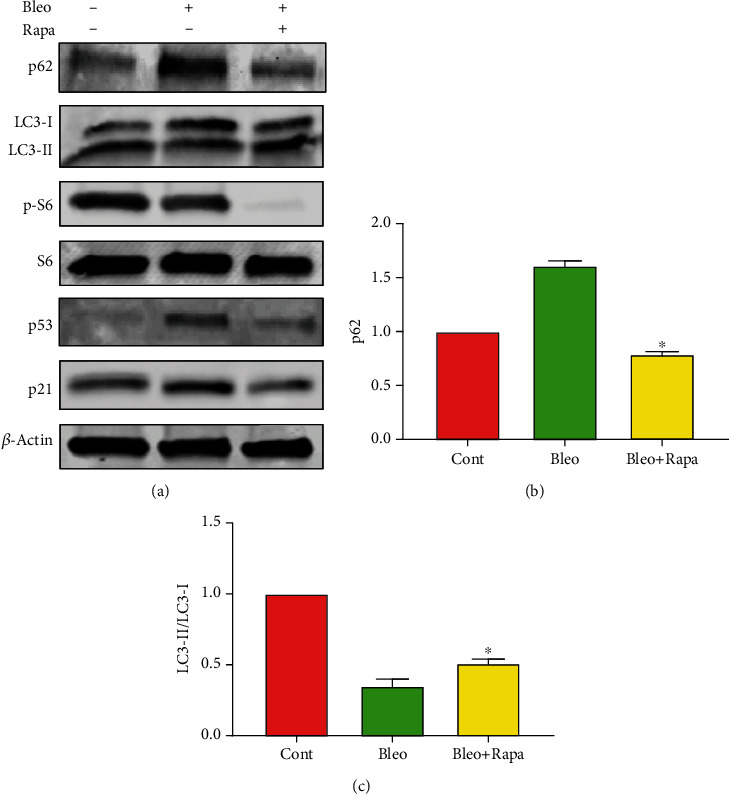
Rapamycin decreases PTSC senescence by upregulating autophagy. (a) Western blot analysis of p62, LC3-II/LC3-I, p-S6, S6, p53, and p21 shows that bleomycin induces LC3I, p62, p53, and p21; however, they are reduced by rapamycin. (b) Relative density of p62 bands. p62, whose level is inversely correlated with autophagy activity, is higher with bleomycin treatment, but rapamycin decreases the p62 expression. (c) LC3 II/LC3 I ratio, an indicator of autophagy activity, is significantly decreased in the bleomycin treated PTSCs, but increased after rapamycin treatment. Each data point represents at least three independent experiments. ^∗^*p* < 0.05, compared to the bleomycin treatment group. Cont: control; Bleo: bleomycin; Rapa: rapamycin.

To further investigate the role of autophagy in the regulation of PTSC senescence and the potential mechanisms involved, we examined the protein expression levels of autophagy markers and SASP markers with bleomycin and rapamycin treatments. First, we found that the expression of p62, which was inversely correlated with autophagy, was increased in the bleomycin-treated PTSCs (Figure 3(a)). The addition of rapamycin reversed the p62 expression to the basal level as in the control group (Figures 3(a) and 3(b)). Meanwhile, as an indicator of autophagy activation [19], the LC3 II/LC3 I expression ratio was significantly reduced by bleomycin treatment in the PTSCs, but rapamycin antagonized this decrease (Figures 3(a) and 3(c)). Moreover, we found that rapamycin at the dose of 25 nM completely inhibited the downstream responder S6 phosphorylation, indicating an inhibition of the mTOR signaling pathway (Figure 3(a)).

When we examined the SASP markers, we found that the expression of p53 was induced by bleomycin treatment, indicating elevated DNA damages. This was accompanied by a slight increase in the p21 expression, confirming the activation of cellular senescence brought by bleomycin. Finally, rapamycin decreased both the p53 and p21 expression in these PTSCs (Figure 3(a)). Taken together, inhibition of PTSC senescence by rapamycin is, at least partially, through the upregulation of autophagy markers.

### 3.4. Rapamycin Promotes Translocation of HMGB1 from the Nucleus to the Cytosol

**Figure 4:**
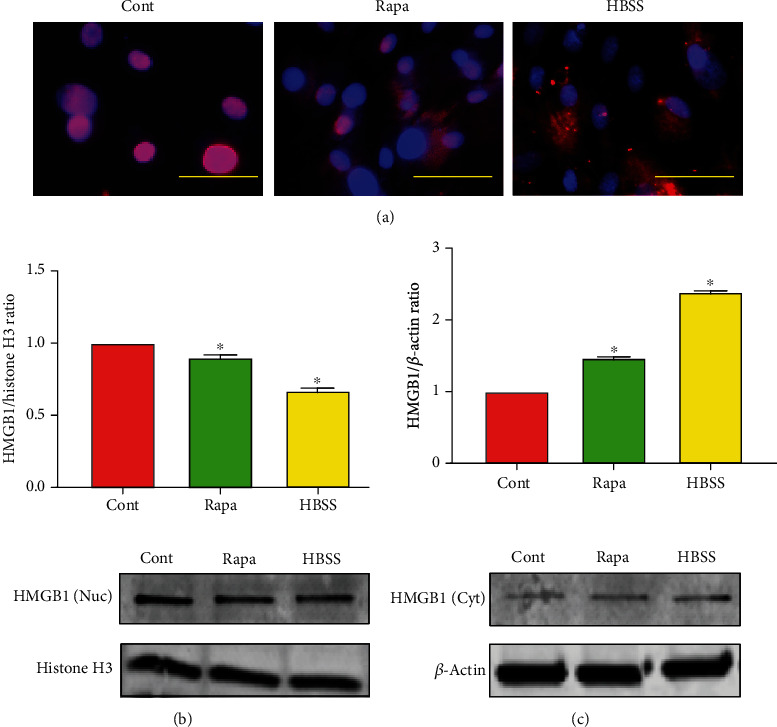
Rapamycin promotes translocation of HMGB1 from the nucleus to the cytosol. (a) HMGB1 translocates from the nucleus to the cytosol after rapamycin and starvation (HBSS) treatment. Rat PTSCs were treated with 25 nM rapamycin for 12 h, starvation (HBSS) for 3 h, then allowed a 12 h recovery, and then immunostained with HMGB1 (red) and Hoechst 33342 (blue). (b, c) Western blot analysis of HMGB1 in the nucleus (Nuc) and cytosol (Cyt) shows that rapamycin reduces nuclear HMGB1 levels but increases cytosol HMGB1 levels. Cells were subjected to nuclear/cytosol fractionation and immunoblotted against HMGB1. Histone H3 and *β*-actin were used as controls. Each data point in (b) and (c) represents at least three independent experiments. ^∗^*p* < 0.05, compared to the untreated group. Cont: control; Bleo: bleomycin; Rapa: rapamycin.

Next, we examined the subcellular expression of HMGB1, which is one of the DAMPs responding to DNA damage stress and a critical regulator of autophagy [28]. When PTSCs were cultured in the standard autophagy inducer HBSS, or treated with rapamycin, HMGB1 clearly translocated from the nucleus to the cytosol, as shown in the immunostaining results (Figure 4(a)). In addition, Western blotting of cellular compartments showed a decrease of HMGB-1 protein levels in the nucleus, with a simultaneous increase of the HMGB1 expression in the cytosol after rapamycin treatment (Figures 4(b) and 4(c)). The similar results obtained from both HBSS and rapamycin treatments indicate a correlation of HMGB1 nucleus-to-cytosol translocation with autophagy activation.

### 3.5. Rapamycin Reduces Catabolic Responses and Inflammation

**Figure 5:**
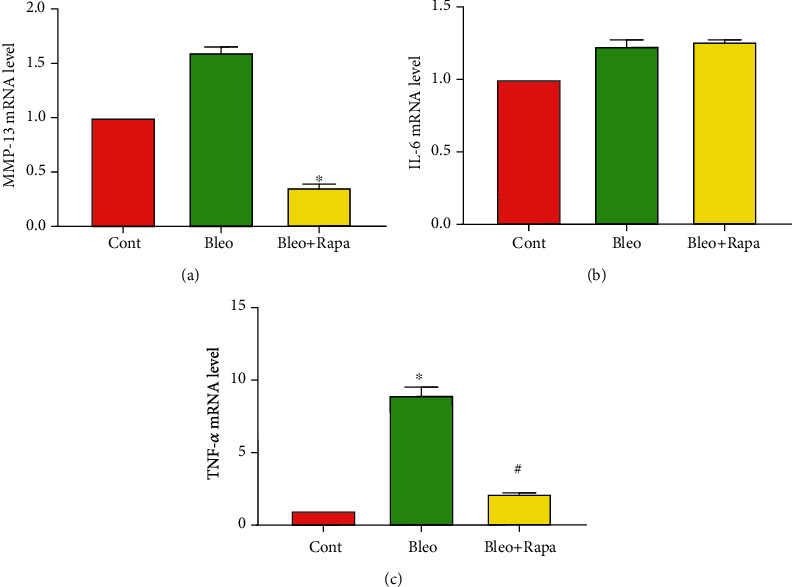
Rapamycin reduces the inflammatory/catabolic response in rat PTSCs. (a) qRT-PCR shows that rapamycin decreases the MMP-13 expression that was upregulated by bleomycin. (b) No significant difference was observed on the IL-6 expression when cells were treated with bleomycin alone, or a combination with rapamycin. (c) Rapamycin decreases the bleomycin-induced TNF-*α* expression. Results are expressed as mean ± SD, *n* = 3. ^∗^*p* < 0.05 compared to control, ^#^*p* < 0.05 compared to the bleomycin treatment group. Cont: control; Bleo: bleomycin; Rapa: rapamycin.

Our previous study has shown that HMGB1 was released into the extracellular matrix when tendon was subjected to excessive mechanical stress, which causes tendon inflammation [34]. Given the active subcellular translocation of HMGB1 induced by autophagy in TSC, as shown above, we next investigated the effects of bleomycin and rapamycin treatments on the expression of tendon inflammation-associated genes. Rat PTSCs treated with bleomycin showed significant upregulation of the catabolic gene MMP-13, which was drastically reduced with rapamycin treatment, compared with the untreated control (Figure 5(a)). There were however no significant differences in the IL-6 gene expression with either bleomycin or rapamycin treatment (Figure 5(b)). Moreover, rapamycin significantly reduced the inflammatory marker TNF-*α* gene expression that was elevated when PTSCs were exposed to bleomycin treatment (Figure 5(c)). Taken together, these results indicated that bleomycin treatment may induce an inflammatory/catabolic response from PTSCs, and such negative response could be reversed, at least partially, by rapamycin treatment.

## 4. Discussion

The number of tendon stem cells and their self-renewal potentials is reduced in elderly tendinopathy patients compared to young patients, leading to a possible role of impaired stem cell potential and differentiation in the tendon structure during aging [35]. The correlation of cellular senescence and age-associated tissue dysfunction has been hypothesized [36]. TSCs from aged/degenerated human Achilles tendon biopsies exhibit proliferation and clonogenicity deficits accompanied by premature entry into cellular senescence by upregulation of p16^Ink4a^. The stem cells become exhausted during tendon aging and degeneration, in terms of size and functional fitness. Sufficient healthy stem cells are essential for tendon tissue regeneration [37]. Our study links the reversal of tendon stem cell senescence to rapamycin, potentially through induction of autophagy. This study may have important implications for preventing cell senescence and aging-induced tendinopathy, as well as for the selection of novel therapeutic targets of chronic tendon diseases.

Our results showed that the treatment of bleomycin, a DNA damaging agent, induced rat PTSC cellular senescence. The senescence was characterized by an increase in the senescence-associated *β*-galactosidase activity, as well as senescence-associated changes in cell morphology. The senescent phenotype induced by bleomycin in PTSCs is also consistent with that in alveolar epithelial cells [11]. On the other hand, rapamycin could extend lifespan in multiple species, including yeast, fruit flies, and mice, by decelerating DNA damage accumulation and cellular senescence [38, 39]. As an inhibitor of mTOR, rapamycin is a prospect of pharmacological rejuvenation of aging stem cells [40]. Our findings show that rapamycin partially decreases the senescence-associated *β*-gal activity and morphological alterations, which indicate that rapamycin reverses senescence in rat PTSCs at both molecular and cellular levels.

Autophagy is a major mechanism for maintaining cellular homeostasis via autophagic cell death. Studies have shown that the activity of autophagy is constitutively high in mesenchymal, hematopoietic, dermal, and epidermal stem cells [13, 41]. Autophagy plays a key role in the control of self-renewal and the stemness of stem cells, and growing evidences have linked autophagy and the mTOR signaling pathway [42, 43]. Some proposed underlying antiaging mechanisms by rapamycin include downregulated translation, increased autophagy, altered metabolism, and increased stress resistance [44]. In this study, we have demonstrated that bleomycin treatment increases the p62 expression, while decreases LC3 II/LC3 I ratio, and rapamycin treatment reverses these molecular changes induced by bleomycin, thus reroutes the senescent TSCs to autophagic signaling. These findings support the idea that the beneficial effects of rapamycin for the TSC senescence might be through the mechanism of autophagy induction.

HMGB1 is a conserved nuclear protein that plays subcellular localization-dependent roles in the regulation of autophagy. It has been found that HMGB1 knockout mice die shortly after birth, and the increased HMGB1 expression inhibits apoptosis in various types of cancer cells [30, 45]. HMGB1 is shown to be present in human tendinopathy, and it can regulate inflammatory cytokines and matrix changes [46]. However, the expression of HMGB1 in the cytosol is a prerequisite for its role to induce autophagy. In this study, we showed that rapamycin promoted HMGB1 translocation from the nucleus to the cytosol, which is directly involved in a positive promotion and maintenance of autophagy in the stressed cells. The secretion of HMGB1 from the PTSCs to the extracellular environment was undetectable (data not shown), suggesting a specific association of HMGB1 to the activation of autophagy rather than inflammation.

Recent evidences have shown that persistent DNA damage could induce the release of inflammatory cytokines, growth factors, and proteases to the extracellular environment [47]. In this study, we found that bleomycin could promote a significant upregulation of the proinflammatory cytokine TNF-*α* and protease MMP-13 without affecting the IL-6 gene expression. This was in line with previous findings that senescent cells excessively increase the expression of secreted molecules that were associated with inflammation, and increased levels of TNF-*α* and MMP-13 can accelerate tendon degeneration in vivo [48, 49]. Therefore, the entry of TSCs into senescence may acquire SASP that accelerates tendon degeneration. Here, we showed that rapamycin dramatically reduced the expression of TNF-*α* and MMP-13, which was elevated with bleomycin treatment, suggesting an inhibition of SASP at the transcript level. Previous studies have shown that MMP-13 increases in human tendinopathy, and increase in MMP-13 in the rat tendon may play a predominant role in matrix degradation following fatigue damage [50, 51]. Therefore, our data might shed some light on potential physiological benefits in tendon matrix integrity brought by rapamycin.

A potential limitation of this study is that we only used a single senescence-inducing stimulus, bleomycin, to induce TSC senescence. However, in the process of organismal aging, individual cells experience multiple cellular pressures, including various kinds of genotoxic, proteotoxic, and mitotic stresses [52]. These stimuli are signaled through various pathways, and the mechanisms that ultimately lead to senescence may also vary depending on the cell type and conditions. Thus, it will be imperative to examine the impact of the mTOR pathway on the various senescence-promoting stressors. Future studies should also address whether rapamycin induces morphological and molecular changes in TSCs of aging tendons.

## 5. Conclusion

This study shows that rapamycin may represent a novel therapeutic to slow down the development of the aging-associated tendinopathy by inhibiting TSC senescence via promoting autophagy. Further studies *in vivo* are needed to confirm the involvement and corresponding mechanisms of mTOR signaling and autophagy in the prevention of aging-induced tendinopathy.

## Data Availability

The data used to support the findings of this study are available from the corresponding author upon request.
